# 

**Published:** 1847-03

**Authors:** 


### CONTENTS.

### LIBRARY.

Page.
Complete Elements of the Science and Art of the Dentist. Translated from the French of M. Desirabode, by *—A—*.................. 193

### JOURNAL.

ART. I.—Valedictory Address before the Baltimore College. By A. Westcott, M. D., D. D. S........ ............................ 201
ART. II.—Inhalation of Ethereal Vapor, for inducing Insensibility to the Pain of Surgical Operations. By A. Hill, D. D. S.............. 224
ART. III.—Insensibility produced by Ethereal Inhalation. By T. E. B. of the Baltimore College.......................... ..... ........ 230
ART. IV.—A new Instrument for remedying Imperfection of Speech, consequent upon Congenital Fissure of the Soft Palate. By C. H. Stearns, Surgeon........................................ 235
ART. V.—Observations on Congential Fissure of the Palate, &c. By C. H. Stearns, Surgeon ..................... .... ......... 238
ART. VI.—Lecture on the Diseases and operations of the Teeth. By Edwin Saunders, Surgeon Dentist................................ 260
ART. VII.—On the Exchange of the Deciduous for the Permanent Incisor Teeth. By Wm. Lintott.................................... 271
ART. VIII.—Uvula Scissors, a New Instrument. By Dr. S. P. Hullihen 274
ART. IX.—Annual Meetings of the Pennsylvania Association of Dental Surgeons.................... ....................... 278

### COLLECTANEA.

Researches on Neuralgia treated by Quinine........................... 277
Remarks on application of Poultices to Inflamed Eyes................ 278
On the manner in which Phosphate of Lime enters Organized Beings..... 279
Fœtus, with left arm partly amputated by the Funis Coiled round it...... 280
Diagnosis of Neuralgia and Neuritis................ ................ 280
A cure of Club Foot and one of Diseases of the Antrum............. ... 281

### MISCELLANEOUS NOTICES.

Baltimore College of Dental Surgery.................................. 282
A Visit to Washington. By Syracuse Editor..................... ..... 284
Forceps for extracting Upper Molar Roots, when not separated.......... 288
Correction. By Syracuse Editor...................................... 289
Works on Operative and Mechanical Dentistry......................... 290
File Carriers........................................................ 291
Arkansas Oil Stone............. ..................................... 293
Introductory Lecture on Materia Medica. By Robert M. Hoston, M. D... 293
Swallowing a "Precious Morsel"—a Plate and Eleven Teeth............. 294
Introductory Lecture on Obstetrics and the Diseases of Women and Children, By Gunning S. Bradford, M. D.............................. 296
Correspondence between the Graduating Class of the Baltimore College and Dr. A. Westcott............................................ 296

